# Spontaneous splenic rupture in Waldenstrom's macroglobulinemia: a case report

**DOI:** 10.1186/1752-1947-4-300

**Published:** 2010-09-08

**Authors:** Michail Charakidis, David Joseph Russell

**Affiliations:** 1 Department of Haematology-Oncology, Royal Hobart Hospital, Tasmania, 7000, Australia

## Abstract

**Introduction:**

We report the case of a patient with Waldenstrom's macroglobulinemia complicated by spontaneous splenic rupture.

**Case presentation:**

A 49-year-old Caucasian woman was referred to our emergency department by her general practitioner following a three-week history of malaise, night sweats, six kilograms of weight loss, intermittent nausea and vomiting, progressive upper abdominal pain and easy bruising. On the fourth day following her admission, she had a rapid clinical deterioration, with subsequent radiological investigations revealing a splenic rupture. Her morphology, biochemistry, flow cytometry and histology were strongly suggestive of Waldenstrom's macroglobulinemia.

**Conclusions:**

Spontaneous splenic rupture is not an expected complication of low-grade lymphoplasmacytic lymphomas, such as Waldenstrom's macroglobulinemia. To the best of our knowledge, this is the only reported case of early spontaneous splenic rupture due to Waldenstrom's macroglobulinemia. Our case highlights that despite the typical disease course of low-grade hematological malignancies, signs and symptoms of imminent splenic rupture should be considered when formulating a clinical assessment.

## Introduction

According to current World Health Organization (WHO) consensus, Waldenstrom's macroglobulinemia (WM) is defined as a lymphoplasmacytic lymphoma (LPL) with bone marrow involvement and an IgM of any concentration [1,2]. Although a familial component has been identified in up to 20 percent of patients, WM is generally considered a sporadic disease [1,2]. The most important risk factor is IgM monoclonal gammopathy of undetermined significance (MGUS). Another implicated epidemiological factor in the development of LPL is concomitant chronic hepatitis C viral (HCV) infection, which is thought to potentially contribute to the pathogenesis of the disease [3].

The presentation of WM is most commonly heralded by the onset of non-specific symptoms, such as weakness and fatigue. As the disease progresses, specific symptoms such as cytopenias, visceral abdominal pain, visual disturbances and peripheral neuropathies become evident. These symptoms reflect tumour infiltration of lymphoid tissues and bone marrow, increased serum immunoglobulin, tissue deposition of IgM, and auto-antibody activity of IgM [1].

Splenic rupture is a well-documented potential complication of high-grade lymphomas and massive splenomegaly, but it is a rare phenomenon in low-grade lymphomas, and previously has not been reported in WM. We report the case of a patient with a spontaneous splenic rupture due to WM.

## Case presentation

A 49-year-old Caucasian woman was referred to our emergency department by her general practitioner regarding a three-week history of generalised malaise, night sweats, weight loss of 6 kg, intermittent nausea and vomiting, progressive upper abdominal pain and easy bruising.

Our observations revealed she had a low-grade fever, blood pressure of 111/81 mmHg, a pulse rate of 80 bpm, and SpO2 of 97 percent on room air. Examination revealed a tender hepato-splenomegaly, with cervical, axillary and inguinal lymphadenopathy.

Our initial investigations demonstrated a normo-chromic-normo-cytic anemia with a hemoglobin of 103 g/L. Leukocytosis of 12.9/nL; with a lymphocytosis of 9.9/nL. Thrombocytopenia of 82/nL, and an elevated erythrocyte sedimentation rate of 31 mm. Her biochemistry showed a total protein of 93 g/L, with a hypoalbuminemia of 29 g/L. A cholestatic picture was suggested on liver function assay, with her alkaline phosphatase and gamma-glutamyl transferase elevated at 142 units/L and 78 units/L respectively. Her lactate dehydrogenase was elevated at 315 units/L and her international normalised ratio was 1.2.

**Figure 1:**
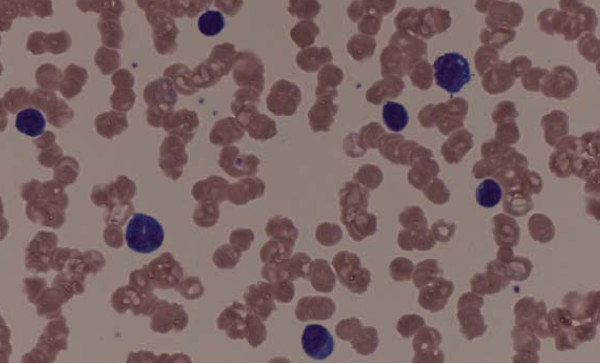
**Medium-power field of peripheral blood smear showing rouleaux formation, small atypical lymphocytes, plasmacytoid cells and mild thrombocytopenia**.

**Figure 2:**
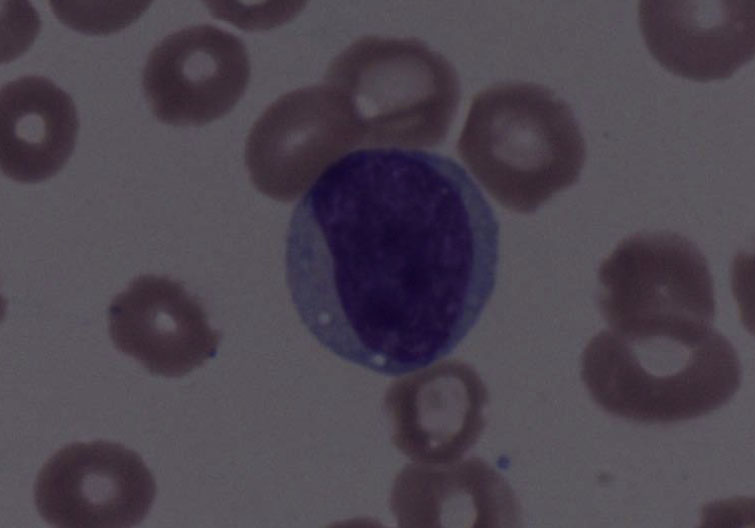
**High-power field of peripheral blood smear revealing a large, atypical B cell with mild cytoplasmic expansion, coarse chromatin, multiple distinct nucleoli and peripheral vacuolation**.

Her peripheral blood smear on medium-power field revealed rouleaux formation, atypical lymphocytes and plasmacytoid cells (figure 1). High-power magnification detected atypical B cells in her peripheral blood with cytoplasmic expansion, coarse chromatin, multiple distinct nucleoli and peripheral vacuolation (figure 2).

**Figure 3:**
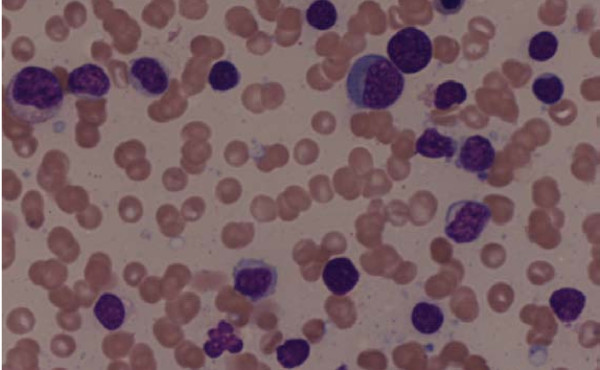
**Medium-power field of bone marrow aspirate demonstrating a population of small atypical lymphocytes admixed with normal cells of erythroid, myeloid and lymphoid lineage**.

**Figure 4:**
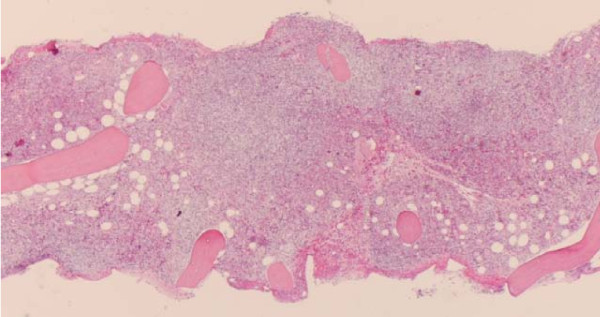
**Hematoxylin and eosin stain of bone marrow trephine identifying large intratrabecular lymphoid aggregates but CD10 negativity excluding follicular lymphoma**.

**Figure 5:**
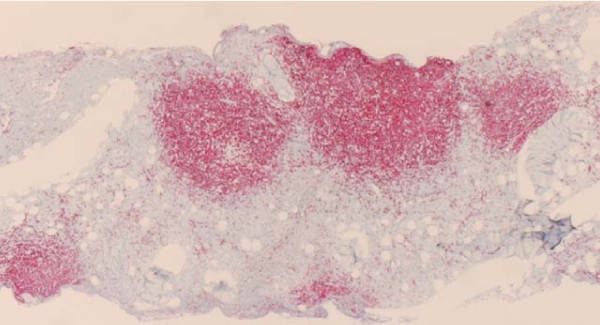
**CD20 stain of bone marrow trephine confirming the strong CD20 positivity of the lymphoid aggregates**.

**Figure 6:**
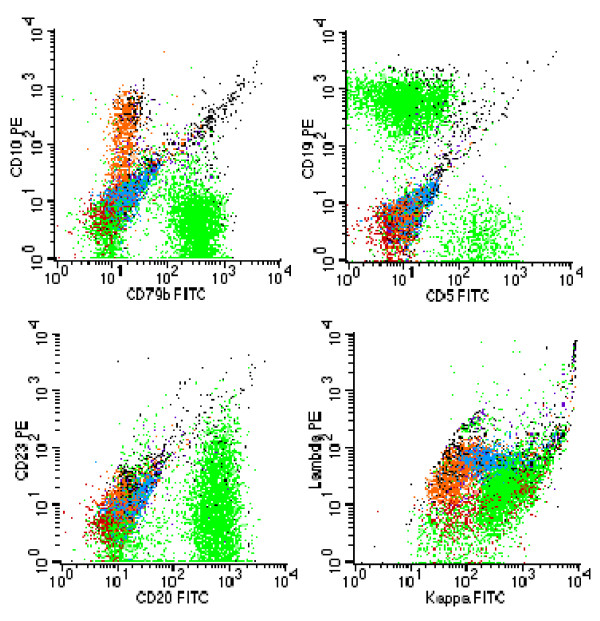
**Flow cytometry of bone marrow demonstrating that 77 percent of lymphoid cells are CD19, CD20, CD79b and cytoplasmic kappa positive**. They are CD5, CD10, CD38 and CD138 negative.

Her bone marrow aspirate demonstrated a population of small atypical lymphocytes admixed with normal cells (figure 3). Interestingly, a hematoxylin and eosin stain of her bone marrow trephine identified large intratrabecular lymphoid aggregates but the absence of CD10 (figure 4). A further stain confirmed strong CD20 positivity of the lymphoid aggregates (figure 5). The flow cytometry of her bone marrow sample demonstrated that 77 percent of the lymphoid cells were CD19, CD20, CD79b, and cytoplasmic kappa positive. They were CD5, CD10, CD38 and CD138 negative (figure 6).

Serum electrophoresis (table 1), appeared to show a large IgM component - more than 30 g/L - in addition to a mild increase in the serum IgG concentration. Immunofixation revealed a monoclonal band that fixes with anti-IgM and anti-kappa. Bence-Jones protein was not detected. Her cytogenetics were 46 XX.

**Table 1:** Serum electrophoresis

Protein	94 g/L	(60-80)g/L
IgA	7.98g/L	(5.5-6.3)g/L

IgG	6.99g/L	(0.65-4.21)g/L

IgM	30.93g/L	(0.3-2.1)g/L

Large IgM component of more than 30 g/L and a mild increase in the serum IgG

**Figure 7:**
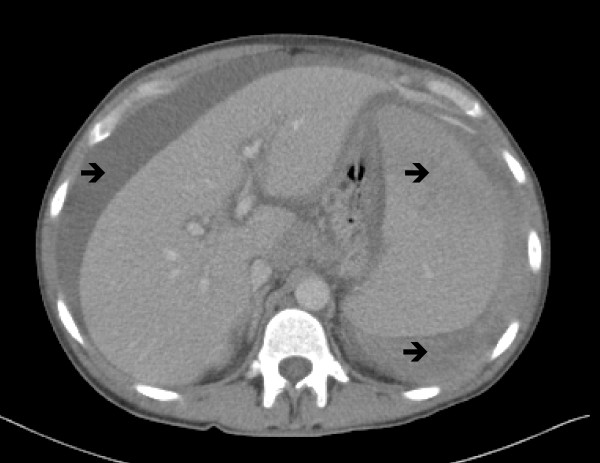
**Abdominal computed tomography (CT) showing significant hemoperitoneum, with extravasation of contrast into the right flank/para-colic gutter**. Hepatomegaly and splenomegaly are clearly seen.

She progressed without event until the fourth day following her admission, when a medical emergency call was initiated for an episode of worsening abdominal pain and hypotension at 88/67 mmHg. Fluid resuscitation was inadequate to maintain her systolic blood pressure at greater than 100 mmHg. An urgent computed tomography (CT) scan illustrated that her spleen was enlarged with a craniocaudal extent of approximately 20 cm, with an extensive hemoperitoneum secondary to an active hemorrhage from a laceration of the superior/anterior pole of her spleen (Figure 7). Para-aortic, celiac axis and porta hepaticus adenopathy was noted, with the largest node measuring 19 mm.

**Figure 8:**
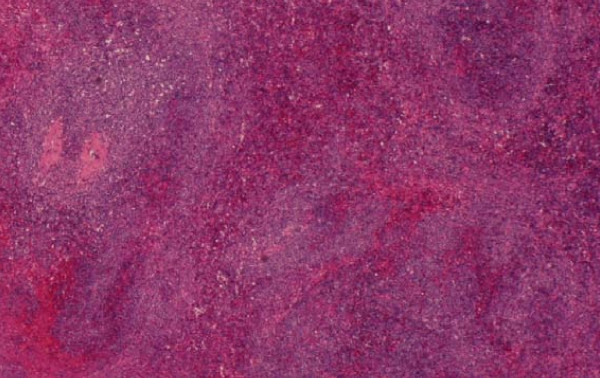
**Low-power magnification of the splenic tissue**. This slide displays significant distortion and diffuse infiltration of the splenic parenchyma by lymphoid cells. Of particular note is the expansion of the white pulp by this infiltrate.

**Figure 9:**
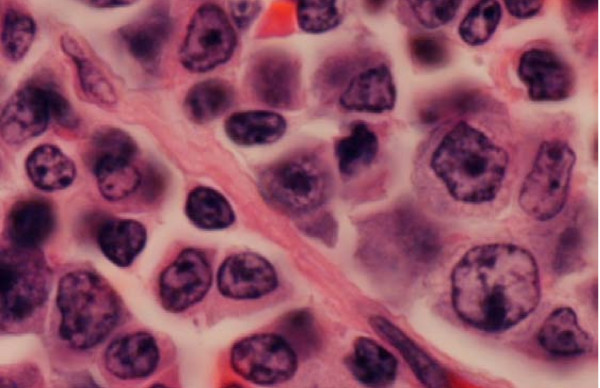
**High-power magnification of splenic lymphoid infiltrate**. This slide demonstrates that the infiltrate consists of small- and medium-sized atypical lymphocytes, which display dense chromatin clumping and prominent nucleoli.

Splenic histology on low-power magnification displayed significant distortion of her splenic tissue and diffuse infiltration by lymphoid cells. There was also expansion of the white pulp by this infiltrate (figure 8). On high-power magnification, we saw infiltrates consisting of small- and medium-sized atypical lymphocytes, which displayed dense chromatin clumping and prominent nucleoli (figure 9).

Finally, in the splenic flow cytometry, the majority of cells gated in the lymphoid region. Approximately 82 percent of lymphocytes were CD19, CD20, CD22 positive, Kappa positive B cells. A total of 25 percent were CD38 and 21 percent CD138 positive.

## Discussion

As mentioned previously an epidemiological link has been established between HCV infection and non-Hodgkin's lymphoma (NHL), (including LPLs), which is especially pronounced in Southern-European, Japanese, and Brazilian populations, conferring an estimated relative risk of 2.5 in the development of any NHL. Marginal zone lymphomas (MZL) are the most commonly-encountered HCV-related lymphoma [3]. Despite this association, the exact pathogenetic role of the virus is not yet clearly established [3].

The 2008 WHO consensus on hematological malignancies bases the diagnosis of WM on a number of findings, including morphology, flow-cytometry, cytogenetics, and biochemistry. With regard to morphology, the predominant features of WM are that of a diffuse infiltration of the bone marrow by small lymphocytes, plasma cells and plasmacytoid cells. Splenic architecture, when available, demonstrates a lymphoplasmacytic infiltrate, composed predominantly of small lymphocytes that may form small nodules in the red pulp or appear more diffusely infiltrated into the splenic parenchyma [2]. Typically the immunophenotype of WM is CD19, CD20, CD79a positivity, with light chain restriction. This is identical to that of our patient. No cytogenetic aberration is specific to WM and the karyotype is usually normal, as opposed to B-cell lymphomas such as splenic MZL and nodal MZL, which demonstrate immunoglobulin heavy and light chain aberrations in many cases [2,4]. Serum electrophoresis demonstrates markedly elevated levels of IgM protein; in addition, reciprocal depression of IgG and IgA can be seen in up to 25 percent of cases [5].

In our case report, an increase in IgG was seen. Immunofixation showed that this was polyclonal and therefore likely to be attributable to the underlying HCV infection. It is of particular note that antiviral therapy directed against HCV infection in the setting of some LPLs has been associated with a favourable prognosis in terms of disease regression [4].

Clinicians are often faced with a diagnostic dilemma when attempting to establish a definitive diagnosis of WM due to the overlap of clinicopathological features with other B-cell lymphomas, including MZL (splenic and nodal), mantle cell lymphoma, small cell lymphocytic lymphoma/chronic lymphocytic leukemia, and even diffuse large B-cell lymphoma. As mentioned previously, the diagnosis of B-cell lymphoma utilizes a combination of both clinical features and a myriad of cellular parameters. In the absence of specific disease markers (such as in WM), this combination of factors is important in attaining a 'pattern of best-fit' when formulating a diagnosis.

Splenic rupture is not an expected sequela of WM. Although infiltration of tissue parenchyma, venous congestion, and IgM-induced coagulopathies are features of WM, they have not yet been reported to lead directly to splenic rupture in the absence of a precipitating event. In our case report, the absence of a previously abnormal spleen, a precipitating traumatic event or a transformation into a diffuse large B-cell lymphoma suggests that our case may represent a more aggressive phenotype of WM.

## Conclusions

Spontaneous splenic rupture is a complication of rapid disease progression, and therefore is not an expected complication of low-grade LPLs, such as WM. To the best of our knowledge, this is the only reported case of early spontaneous splenic rupture due to WM.

Our case report highlights that, despite the typical disease course of low-grade hematological malignancies, signs and symptoms of imminent splenic rupture should be considered when formulating a clinical assessment.

## Consent

Written informed consent was obtained from the patient for publication of this case report and any accompanying images. A copy of the written consent is available for review by the Editor-in-Chief of this journal.

## Competing interests

The authors declare that they have no competing interests.

## Authors' contributions

MC and DJR are the sole authors and contributed equally to the production of this manuscript. All authors read and approved the final manuscript.
